# Gender Differences in Plasma Biomarker Levels in a Cohort of COPD Patients: A Pilot Study

**DOI:** 10.1371/journal.pone.0016021

**Published:** 2011-01-18

**Authors:** Juan P. de Torres, Ciro Casanova, Victor Pinto-Plata, Nerea Varo, Patricia Restituto, Elizabeth Cordoba-Lanus, Rebeca Baz-Dávila, Armando Aguirre-Jaime, Bartolome R. Celli

**Affiliations:** 1 Pulmonary Department, Clínica Universidad de Navarra, Pamplona, Spain; 2 Pulmonary Department, Hospital Universitario Nuestra Señora de Candelaria, Santa Cruz de Tenerife, Spain; 3 Pulmonary and Critical Care Department, St Elizabeth's Medical Center, Boston, Massachusetts, United States of America; 4 Biochemical Analysis Department, Clínica Universidad de Navarra, Pamplona, Spain; 5 Respiratory Research Unit, Hospital Universitario Nuestra Señora de Candelaria, Santa Cruz de Tenerife, Spain; 6 Pulmonary Department, Brigham and Women's Hospital, Harvard Medical School, Boston, Massachusetts, United States of America; University of Alabama-Birmingham, United States of America

## Abstract

**Rationale:**

Little is known about gender differences in plasma biomarker levels in patients with chronic obstructive pulmonary disease (COPD).

**Hypothesis:**

There are differences in serum biomarker levels between women and men with COPD.

**Objective:**

Explore gender differences in plasma biomarker levels in patients with COPD and smokers without COPD.

**Methods:**

We measured plasma levels of IL-6, IL-8, IL-16, MCP-1, MMP-9, PARC and VEGF in 80 smokers without COPD (40 males, 40 females) and 152 stable COPD patients (76 males, 76 females) with similar airflow obstruction. We determined anthropometrics, smoking history, lung function, exercise tolerance, body composition, BODE index, co-morbidities and quality of life. We then explored associations between plasma biomarkers levels and the clinical characteristics of the patients and also with the clinical and physiological variables known to predict outcome in COPD.

**Results:**

The plasma biomarkers level explored were similar in men and women without COPD. In contrast, in patients with COPD the median value in pg/mL of IL-6 (6.26 vs 8.0, p = 0.03), IL-16 (390 vs 321, p = 0.009) and VEGF (50 vs 87, p = 0.02) differed between women and men. Adjusted for smoking history, gender was independently associated with IL-16, PARC and VEGF levels. There were also gender differences in the associations between IL-6, IL-16 and VEGF and physiologic variables that predict outcomes.

**Conclusions:**

In stable COPD patients with similar airflow obstruction, there are gender differences in plasma biomarker levels and in the association between biomarker levels and important clinical or physiological variables. Further studies should confirm our findings.

## Introduction

The prevalence of tobacco-related diseases in women, mainly chronic obstructive pulmonary disease (COPD) and lung cancer continues to increase [1]. In the Western world, the bulk of the increase in COPD related morbidity and mortality is driven by the increasing burden of disease in women [2] likely related to the historic increase of smoking among women in these areas [3].

Compared with men with similar degree of airflow obstruction, women with COPD have worse dyspnea and quality of life scores, lower body mass index (BMI) and fat free mass (FFM) [4], and manifest a higher prevalence of anxiety and depression [5]. In spite of these differences, they appear to have a better survival [6].

Several plasma biomarkers differ in their concentration between patients with COPD and normal controls [7]. The serum level of these biomarkers (CRP, IL-6, TNFα, MMP-9, PARC, VEGF) have been associated with some of the clinical features of the disease [8], [9] and have been proposed to be related to the systemic consequences of the disease [10], its progression [11] and the association between COPD with cardiovascular disease [12] and lung cancer [13].

In spite of the differences in the clinical expression of COPD between women and men, surprisingly little is known about gender differences in plasma levels of biomarkers in patients with the disease. The identification of different biological response in men and women to the factors known to cause COPD could help us understand some of the different aspects of the clinical presentation of the disease and perhaps guide us in the development of gender specific research and possible therapy.

We hypothesized that compared with men; women with COPD have different levels of plasma biomarkers known to be abnormal in patients with COPD. To test this hypothesis we conducted a pilot study in a selected population of smokers without airflow obstruction and a cohort of COPD patients attending pulmonary clinics. Both groups consisted of equal number of men and women of similar age and lung function. Based on our previous work [6], we selected 8 markers representing different biological pathways that also showed strong association with COPD. As markers of inflammation we measured the interleukins 6, 8, and16 (IL-6, IL-8, IL-16), tumor necrosis factor alpha (TNF α). Vascular endothelial growth factor (VEGF) and matrix metalloproteinase nine (MMP-9) were selected as markers of injury and repair. Monocyte chemoattractant protein-1 (MCP-1/CCL2), and pulmonary activation regulated protein (PARC/CCL-18) were selected as primary chemoattractants. The level of these markers also relates to clinical variables of importance in COPD.

## Methods

Smokers without COPD and patients with COPD older than 40 years old were invited to participate in the study. The COPD patients were part of the BODE observational study, recruited between January 1997 and December 2008. The patients that participated in this study were recruited in pulmonary clinics in two tertiary university hospitals in the United States (St. Elizabeth's Medical Center, Boston) and Spain (Nuestra Sra de La Candelaria, Tenerife) [14].

COPD was defined by a history of smoking at least 10 pack-years and a FEV_1_/FVC ratio less than 0.7 measured 20 minutes after the administration of 400ug of inhaled albuterol. Patients were excluded if they had a history of asthma, bronchiectasis, tuberculosis or other confounding diseases like severe congestive heart failure (Stage II through IV NYHA), obliterative bronchiolitis or diffuse panbronchiolitis. All COPD participants were clinically stable (free of exacerbation for at least 8 weeks) and were receiving standard medical treatment according to the ATS/ERS consensus [15].

Healthy smokers, defined by a history of smoking at least 10 pack-years and a FEV_1_/FVC ratio of more than 0.7 measured 20 minutes after the administration of 400ug of inhaled albuterol, were also recruited in both medical centers. They did not have known history of asthma or any other history of pulmonary disease.

The human-research review board at each site (IRB n° 258 Hospital Universitario Ntra Sra de Candelaria and IRB n° 00195 St Elizabeth's Caritas Medical Center IRB committees) approved the study and all patients signed the informed consent.

### Clinical parameters measurements

A personal interview with trained personnel recorded the following information: age, smoking status, pack-years history and co-morbidities using the Charlson scale [16] where points are assigned to the presence of different co-morbidities. The score ranges from 0 to 37, with the higher score implying more co-morbidity.

Pulmonary function [14]and the six minute walking distance (6MWD) were performed following ATS guidelines [17]. Briefly, the 6MWD chosen was the better of two 6-minute walk distance tests separated by 30 minutes in a 30 meters corridor. The inspiratory capacity (IC) was measured as previously described and the IC/TLC was calculated from the lung volume measurements [18].

Dyspnea was assessed using the Modified Medical Research Council Dyspnea Scale (MMRC) [19]. The BMI was calculated as the weight in kilograms divided by height in meters^2^. Arterial blood gases were measured at rest. The BODE index was measured as previously described [13].

We utilized the same matching method for the COPD patients as in other published studies [4], [6]. We selected and matched the patients from an initial sample of 138 males and 76 females with COPD; from this cohort we were able to match every female patient with a male with FEV_1_ % of predicted within ±2%. When more than one male was found to match a female, we randomly chose the patient to be included in the final sample, being blind to the rest of the evaluated parameters.

### Biochemical analyses

Plasma of all study participants was collected into Vacutainer® tubes. Blood specimens were centrifuged (2000×g), and aliquots stored at −80°C until laboratory analysis.

Plasma levels of IL-6, IL-8, IL-16, MCP-1, MMP-9, PARC and VEGF were measured by ELISA (R&D System, Minneapolis) according to the manufacturer's instructions. The detection limits were 0.7 pg/mL, 3.5 pg/mL, 6.2 pg/mL, 5,0 pg/mL, 156 pg/mL, 10 pg/mL, and 5.0 pg/mL, respectively. The within-assay coefficient of variation for all assays was less than 10%.

### Statistical analysis

Quantitative measurements with parametric and non-parametric distribution are presented as mean ± SD or median 25–75^th^ percentiles, while categorical variables are presented as percentages. Sex differences were explored using Student t test, Man-Whitney U tests and Chi square test respectively. To compare sex differences in plasma marker levels we used ANOVA test to adjust for their pack-year history. A multiple linear regression model determined those factors that influence each marker level. We included in the model those factors that showed statistical difference in the univariate analysis (DLCO and pack-years history) and those that demonstrated associations with plasma levels (age, gender, BMI, FEV_1_%). The statistical analyses were adjusted for multiple comparisons. Significance was established as two-tailed p-value≤0.05. Calculations were made with the statistical package SPSS Inc version 15.0 (Chicago, IL, USA).

## Results

The clinical characteristics of the 80 current or former smokers without COPD (40 males and 40 females) are shown in Table 1. No gender differences were found in all clinical parameters evaluated except that the smoking history was higher in men than women.

**Table 1:** Clinical characteristics of smokers without COPD of both genders.

Smokers without COPD			
Clinical Characteristics	Males n = 40	Females n = 40	p Value
**Age yrs** *	63±7	62±7	ns
**BMI Kg/m^2^** *	27±6	27±4	ns
**Smoking Status active**	52%	60%	ns
**Pack-years History** **	44; 38–73	26; 20–50	0.042
**Charlson** **	0;0-0	0;0-0	ns
**FEV_1_%** *	97±16	91±15	ns
**FVC%** *	100±15	96±14	ns

BMI, body mass index. FEV_1_%, forced expiratory volume in one second (% predicted).

FVC%, forced vital capacity (% predicted).

ns = non significant.

*mean±SD.

**median; 25–75^th^ percentiles.

Of the 152 stable COPD patients (76 men and 76 women), 44% of patients were in GOLD stages I and II and 56% in III and IV. Their clinical and physiological characteristics are shown in Table 2. Males and females had similar age, BMI, FEV_1_% FVC%, TLC%, PaCO_2_, PaO_2_ and BODE indexes. Statistical significant differences were found in IC/TLC, DLCO, 6MWD, MMRC and smoking history.

**Table 2:** Clinical characteristics of patients with COPD.

COPD patients			
Clinical Characteristics	Males n = 76	Females n = 76	p Value
**Age yrs** *	64±7	64±8	ns
**BMI Kg/m^2^**	27±4	27±5	ns
**Smoking Status active**	33%	42%	ns
**Pack-years history** *	69±32	57±33	0.008
**Charlson** **	0;0–1	0;0–1	ns
**MMRC** **	1;0–2	2;1–3	0.033
**6MWD m** **	464;378–553	404;335–487	0.012
**FEV_1_%** *	50±21	50±22	ns
**FVC%** *	82±22	79±24	ns
**TLC%** *	117±21	121±23	ns
**IC/TLC** *	0.33±0.09	0.29±0.11	0.015
**DLCO** **	68;44–90	50;35–77	0.007
**PaO_2_ mmHg** *	70±12	72±12	ns
**PaCO_2_ mmHg** *	44±7	43±6	ns
**BODE** *	2;1–4	3;1–5	ns

BMI, body mass index. FEV_1_%, forced expiratory volume in one second (% predicted).

FVC%, forced vital capacity (% predicted). MMRC, modified medical research council dyspnea scale. 6MWD, six-minute walk distance. TLC, total lung capacity.

IC, inspiratory capacity. BODE, body mass index, obstruction, dyspnea and exercise capacity index.

ns = non significant.

*mean±SD.

**median; 25–75^th^ percentiles.

Table 3 shows the plasma biomarker levels in healthy smokers and in COPD patients, adjusting for smoking history. No statistical differences were found in the plasma biomarker levels in healthy men and women smokers. In patients with COPD there was a statistically significant difference in IL-6, IL-16 and VEGF levels between women and men. In the multivariate analysis, gender was a statistical significant predictor of IL-16, PARC and VEGF levels.

**Table 3:** Plasma protein levels in smokers without COPD and COPD.

Marker	IL-6	IL-8	IL-16	MCP-1	MMP-9	PARC	VEGF
**Smokers without COPD**							
**Females**	9.86.9–13.7	7.64.1–11.5	386304–511	395296–632	44853766–5885	3925430954–51759	38.021.0–76.0
**Males**	8.56.9–10.0	5.02.9–7.2	495398–647	521331–636	36703175–4270	3996129015–46794	30.920.1–43.7
**p Value**	ns	ns	ns	ns	ns	ns	ns
**COPD patients**							
**Females**	6.23.5–10.7	9.66.8–13.1	390296–598	502364–765	39402997–15262	4629127725–57780	50.326.8–110.6
**Males**	8.081.9–23.3	9.26.3–15.9	321226–459	511404–648	51743182–14569	4840033863–64697	87.733.0–171.0
**p Value**	0.032	ns	0.009	ns	ns	ns	0.022

Levels values are shown as median in pg/dL with their 25–75^th^ percentiles.

ns = non significant.

Table 4 shows the association between plasma markers that showed gender differences and selected clinical parameters. Plasma levels of IL-6, IL-16 and VEGF had different strength of associations with the clinical variables in the two genders.

**Table 4:** Correlation coefficients for the association between plasma protein levels that showed gender differences and clinical and physiological measurements.

Marker	IL-6	IL-16	VEGF
**Females**			
rSpearman		BMI 0.23	Age−0.31BODE−0.22
**Males**			
rSpearman	DLCO0.27		IC/TLC0.29DLCO0.26BODE−0.25

Only significant values are shown.

## Discussion

The present work has three main findings. First, there are no gender differences in plasma biomarker levels in smokers without COPD with similar clinical characteristics. Second, there are gender differences in some plasma biomarker levels in patients with COPD and similar degree of airflow obstruction. Third, there also gender differences in the strength of the association between selected biomarkers and clinical variables known to be associated with poor outcomes.

Several lines of evidence suggest the presence of gender differences in plasma inflammatory cytokines levels in health [20] as well as in disease [3]. Several factors have been implicated as possible causes for these differences. The most important factors thought to account for these differences include a difference in the proportion of fat tissue and its distribution [20], and the level of sex hormones [21]. These reported differences could influence the way women with respiratory diseases cope with the inflammatory response related to the disease and ultimately relate to the development of lung cancer [22] and COPD [4], [6], [23].

In a well-characterized population of patients with COPD, our group has previously shown that there are important gender differences in the clinical presentation as well as in the survival of men and women with COPD [4], [6]. Based on these clinical findings, we planned this cross sectional observational study to test the hypothesis that COPD patients with similar degree of airflow obstruction manifest gender related differences in plasma biomarkers. The biomarkers were selected based on the results of a previous study by our group using high throughput proteomic analysis [9]. The final grouping included: A. inflammatory markers (IL-6, IL-16, and IL-8); B. Injury and repair markers (MMP-9, VEGF) and C. markers that are thought to be chemoattractants (MCP-1, PARC).

The first observation in this study is that smokers without COPD with similar clinical characteristics did not show gender differences in the plasma levels of the markers measured. This is an important and novel finding because it suggests that there are no gender differences in those smokers that are able to handle the injurious local and systemic response induced by tobacco consumption once age, BMI, smoking status and co-morbidities are controlled. Perhaps this explains the difference with previous reports in healthy individuals where the volunteers had differences in the baseline characteristics [21].

In contrast to the findings in non- COPD controls, we observed that in patients with COPD there are statistically significant gender differences in IL-6, IL-16 and VEGF. Interestingly, different associations with important clinical or physiological outcomes were found with the same biomarkers in each sex, supporting a true gender role.

IL-6 is a pro-inflammatory cytokine that provides a link between innate and acquired immunity. Increased levels of IL-6 in exhaled breath condensate, sputum and plasma has been described in patients with COPD. IL-6 is responsible for stimulating hepatocytes to secrete C reactive protein and has been proposed to be responsible for the systemic consequences of COPD (insulin resistance, osteoporosis [24], muscle degradation [25], and depression [26]). The plasma IL-6 levels were significantly lower in females with COPD compared with smoking women without COPD (p = 0.01). This was not observed in males were the serum level in both groups were similar (p = 0.15). In addition, the plasma IL-6 levels in COPD patients was significantly lower in females than males. The clinical significance of the difference is difficult to ascertain because this has not been determined yet and there is variability in the serum level of this biomarker.

IL-16 is an important chemokine that regulates recruitment and activity of CD4 lymphocytes. Little information is available on plasma levels of IL-16 in COPD patients. It has been related to the process of pulmonary vascular remodeling [27]. Females in our cohort had higher plasma IL-16 levels. The level of IL-16 was associated with BMI in female COPD patients, but the clinical relevance of this finding is unclear. However, this gender difference should be taken in account when this cytokine is analysed in COPD patients.

VEGF has an important role in regulating the growth of new vessels and vascular leakage, and has been shown to be reduced in the lung and sputum of COPD patients. In rats, blockade of VEGF receptor 2 (VEGF-R2) induces alveolar cell wall apoptosis and the development of emphysema like pathology [28]. Previous studies have shown elevated plasma levels in COPD inversely associated with the degree of airway obstruction [9]. In the present work we also found elevated levels of VEGF in COPD in comparison with smokers without COPD. We also observed that gender was one of the predictors of its plasma levels. Its levels correlated with the BODE index in both genders and with markers of lung hyperinflation (IC/TLC) and emphysema (DLCO) in men. These observations suggest a different sex response in this important growth factor that has been implicated in the development of emphysema, a phenotype more prevalent in males [28].

There are some limitations in our study. First, we did not analyze other important plasma markers associated with COPD (CRP, TNF-alpha, surfactant protein D or fibronectin). We chose our panel of plasma markers based on our group's experience, biological plausibility and attempting to represent the multidimensional pathological process of COPD [9]. We also acknowledge that not all phenotypes of the disease are represented in this sample of COPD patients and that identification of phenotypes such as emphysema or chronic bronchitis could help in determining a distinct plasma marker profile. However, our main goal was to explore if gender could influence the level of plasma response and not to comprehensively describe the plasma markers of COPD. Although we did not find significant differences in plasma markers levels in smokers without COPD, we acknowledge this could be due to our sample size, which was relatively small, thereby generating a β type II error. However, we still believe that our sample of healthy smokers (40 males and 40 females) with similar age, BMI and comorbidities, is a good sample to represent normal individuals.

In summary, there were no gender differences in selected plasma biomarker levels in smokers without COPD, while in patients with COPD there were gender differences in some biomarker levels, especially those thought to be implicated in the genesis of emphysema. Further, the gender differences in plasma levels are in line with the reported gender specific clinical manifestations of COPD. We acknowledge this to be a pilot study that should be taken as hypothesis generating work. Further studies measuring these and other plasma markers should confirm the relevance of these findings.

## References

[1] Jamal A, Ward E, Hao Y (2005). Trends in the leading causes of death in the United States, 1970–2002.. JAMA.

[2] Mannino DM, Homa DM, Akinbami LJ, Ford ES, Redd SC (2002). Chronic Pulmonary Disease Surveillance- United States, 1971–2000.. MMWR.

[3] Becklake MR, Kauffmann F (1999). Gender differences in airway behaviour over the human life span.. Thorax.

[4] de Torres JP, Casanova C, Hernandez C, Abreu J, Aguirre-Jaime A (2005). Gender and COPD in patients attending a pulmonary clinic.. Chest.

[5] Di Marco F, Verga M, Reggente M, María Casanova F, Santus P (2006). Anxiety and depression in COPD patients: The roles of gender and disease severity.. Respir Med.

[6] de Torres JP, Cote CG, López MV, Casanova C, Diaz O (2009). Sex differences in Mortality in Patients with COPD.. Eur Respir J.

[7] Gan WQ, Man SF, Senthiselvan A, Sin DD (2004). Association between chronic obstructive pulmonary disease and systemic inflammation: a systematic review and meta-analysis.. Thorax.

[8] de Torres JP, Cordoba-Lanus E, López-Aguilar C, Muros de Fuentes M, Montejo de Garcini A (2006). C-reactive protein levels and clinically important predictive outcomes in stable COPD patients.. Eur Respir J.

[9] Pinto-Plata V, Toso J, Lee K, Park D, Bilello J (2007). Profiling serum biomarkers in patients with COPD: associations with clinical parameters.. Thorax.

[10] Agustí AGN, Noguera A, Sauleda J, Sala E, Pons J (2003). Systemic effects of chronic obstructive pulmonary disease.. Eur Resp J.

[11] Higashimoto Y, Iwata T, Okada M, Satoh H, Fukuda K (2009). Serum biomarkers as predictors of lung function decline in COPD.. Respir Med.

[12] Sin DD, Man SF (2003). Why are patients with chronic obstructive pulmonary disease at increased risk of cardiovascular diseases? The potential role of systemic inflammation in chronic obstructive pulmonary disease.. Circulation.

[13] Houghton AM, Mouded M, Shapiro SD (2008). Common origins of lung cancer and COPD.. Nat Med.

[14] Celli BR, Cote C, Marin JM, Casanova C, Montes de Oca M (2004). The Body Mass Index, Airflow Obstruction, Dyspnea, Exercise Performance (BODE) Index in Chronic Obstructive Pulmonary Disease.. N Engl J Med.

[15] Celli BR, MacNee W (2004). Standards for the diagnosis and treatment of patients with COPD: a summary of the ATS/ERS position paper.. Eur Respir J.

[16] Charlson M, Szatrowsky T, Peterson J, Gold J (1994). Validation of a combined comorbidity index.. J Clin Epidemiol.

[17] ATS Statement: Guidelines for the Six-Minute Walk Test (2002). Am J Respir Crit Care Med.

[18] Casanova C, Cote C, de Torres JP, Aguirre-Jaime A, Marin JM (2004). The Inspiratory to Total Lung Capacity Ratio Predicts Mortality in Patients with COPD.. Am J Respir Crit Care Med.

[19] Brooks SM (1982). Surveillance for respiratory hazards.. ATS News.

[20] Cartier A, Côte M, Lemieux I, Pérusse L, Tremblay A (2009). Sex differences in inflammatory markers: what is the contribution of visceral adiposity.. Am J Clin Nutr.

[21] Ben-Zaken Cohen S, Paré PD, Paul Man SF, Sin DD (2007). The growing burden of Chronic Obstructive Pulmonary Disease and lung Cancer in Women. Examining sex differences in cigarette smoke metabolism.. Am J Respir Crit Care Med.

[22] Henschke CI, Yip R, Miettinen OS (2006). Women's susceptibility to tobacco carcinogens and survival after diagnosis of lung cancer. International Early Lung Cancer Action Program Investigators.. JAMA.

[23] Xu X, Li B, Wang L (1994). Gender difference in smoking effects on adult pulmonary function.. Eur Respir J.

[24] Bolton CE, Ionescu AA, Shiels KM, Pettit RJ, Edwards PH (2004). Associated loss of fat-free mass and bone mineral density in chronic obstructive pulmonary disease.. Am J Respir Crit Care Med.

[25] Di Marco F, Verga M, Reggente M, Casanova MF, Santus P (2006). Anxiety and depression in COPD patients: The roles of gender and disease severity.. Respir Med.

[26] Barnes PJ (2009). The cytokine network in Chronic Obstructive Pulmonary Disease.. Am J Respir Cell Mol Biol.

[27] Conti P, Kempuraj D, Kandere K, Di Gioacchino M, Reale M (2002). Interleukin-16 network in inflammation and allergy.. Allergy Asthma Proc.

[28] Marwick JA, Edirisinghe I, Arunachalam G, Stevenson CS, Macnee W (2010). Cigarette smoke regulates VEGFR2-mediated survival signaling in rat lungs.. J Inflamm (Lond).

